# Rapid detection of *Escherichia coli* in beverages using genetically engineered bacteriophage T7

**DOI:** 10.1186/s13568-019-0776-7

**Published:** 2019-04-19

**Authors:** Nicharee Wisuthiphaet, Xu Yang, Glenn M. Young, Nitin Nitin

**Affiliations:** 1 Department of Food Science and Technology, University of California, Davis, CA USA; 2 Department of Biological and Agricultural Engineering, University of California, Davis, CA USA

**Keywords:** Engineered bacteriophage, *Escherichia coli* BL21, Alkaline phosphatase, ELF-97, Fluorescent microscope, Image analysis

## Abstract

Foodborne illness due to bacterial contamination is a significant issue impacting public health that demands new technology which is practical to implement by food industry. Detection of bacteria in food products and production facilities is a crucial strategy supporting food safety assessments. Bacteriophages were investigated as a tool for bacterial detection due to their ability to infect specific strain of host bacteria in order to improve sensitivity, specificity, and rapidity of bacterial detection. The results of this investigation reveal a novel method for rapid detection. The method employs a genetically engineered bacteriophage, phage T7-ALP, which expresses alkaline phosphatase. Upon infection of *Escherichia coli*, overexpression of alkaline phosphatase provides an opportunity for rapid sensitive detection of a signal indicative of bacterial presence in model beverage samples as low as 100 bacteria per gram. The method employs a fluorescent precipitated substrate, ELF-97, as a substrate for alkaline phosphatase activity coupled with fluorescence imaging and image analysis allowing single-cell imaging results in high detection sensitivity. The method is easily completed within less than 6 h enabling it to be deployed within most large industrial food processing facilities that have routine 8-h operational shifts.

## Introduction

Over the past decades, foodborne disease outbreaks affect millions of people worldwide by causing burdens on public health and significant hindrance in socio-economic development (WHO 2015). Each year, in the United States, approximately 9.4 million cases of foodborne illness were reported causing over $15.5 billion economic burdens (Scallan et al. 2011; Hoffmann et al. 2015), including 64% of the foodborne-related deaths were caused by infection of bacteria (Scallan et al. 2011). Given that it is well-documented the leading causes of foodborne illnesses are due to contamination by bacterial pathogens, detection of these microbes in food is a critical step that ensures the safety of food and beverages products before distribution to the consumers. Among all of these pathogens, *Escherichia coli* is probably one of the most prevalent pathogens during the past few years which resulted in foodborne illness outbreaks. According to the Centers for Disease Control and Prevention (CDC) surveillance of foodborne outbreaks in the U.S., several food products have been reported associated with *E. coli* contamination such as romaine lettuce, ground beef, and beverages including coconut water an apple juice (Marder et al. 2017). Therefore, detection of *E. coli* that is applicable in variety of food matrices is highly crucial.

Culture-based detection methods remain the “gold standard” for pathogen detection in food and animal feed because they provide highly accurate results; however, the major drawback of these methods is that they require 3-5 days to complete and are relatively expensive for total material and labor costs (Koyuncu and Haggblom 2009; Kralik and Ricchi 2017). Economic costs of extended production storage to allow testing and loss of food quality—for short shelf life foods—further negatively impact food manufacturers. Molecular-based methods, such as those employing polymerase chain reaction (PCR) provide alternative opportunities for rapid pathogen detection of bacterial pathogens. Even though PCR offers the results with only few hours of bacteria enrichment, the critical drawback of detection methods relying on PCR is that they are incapable of differentiating between viable and dead cells which leads to false-positive results (Chapela et al. 2015; Kralik and Ricchi 2017). Economically, PCR-based methods remain accessible only to large food manufactures that have the capacity to support the technical application of this approach and the relatively high costs. On a technical level, wide use of PCR-based methods suffer from the complexity of food matrices, many of which have been demonstrated to harbor molecules that are inhibitors of PCR, resulting in decreased sensitivity or false-negative results (Rossen et al. 1992; Schrader et al. 2012). Immunological-based methods, including variations of enzyme-linked immunosorbent assays (ELISAs), have been developed in order to provide more rapid, economical and simple approach compared to the culture-based and PCR-based methods; however, on a practical level, ELISAs suffer from an inability to distinguish live from dead bacteria, from low sensitivity due to small sample volume capacity, and from low specificity due to cross-reactivity of polyclonal antibodies (Ivnitski et al. 1999; Shen et al. 2014). The limitations of these foodborne pathogen detection methods provide incentive for continued research and development into innovative novel approaches for rapid detection that have high sensitivity and specificity to detect bacterial foodborne pathogens, and can be widely adapted for use by a diversity of large and small food manufactures.

Bacteriophages or phages have drawn the attention of many researchers in the field of pathogen detection due to their high specificity to bacterial host strain which enables them to be developed as a tool for detection of specific bacterial pathogen. Phages are also capable of self-replication and produce progeny phages within the short period of time, this allows amplification of the signal intensity resulting in lower limit of detection without the requirement for any overnight enrichment (Anany et al. 2017). One significant advantage of using phages for bacterial detection is the ability to distinguish between viable and dead cells. Since phage infection and replication only occur in living bacterial cells, bacteriophage-based detection methods inherently ensure that only viable cells are targeted (Hagens and Loessner 2007). Several strategies have been employed to develop phage-based biosensors including detection of the released cell components due to phage lysis (Griffiths 1996; Chang et al. 2002; Chen et al. 2015), detection of labeled phages after their specific attachment to bacterial hosts (Hennes et al. 1995; Yim et al. 2009), and detection of the progeny phage amplification using nucleic acid–based detection techniques (González-Gil et al. 1998; Reiman et al. 2007; Kutin et al. 2009).

Recently, genetically engineered phages have been created in order to provide special features that allow greater possibilities in developing novel bacterial biosensors. Among the most promising approaches is ‘Reporter Phage’, where wild-type phages are genetically modified to harbor reporter genes that can only be activated once phage-host infection occurs. Expression of reporter genes inside host cells yields a detectable signal indicative of the presence of target bacteria (Smartt et al. 2012; Singh et al. 2013). Several gene-based reporters have been widely studied and incorporated into phage genome, such as *lux* locus for bioluminescence (Loessner et al. 1997; Kim et al. 2017), *lacZ* encoding β-galactosidase (Goodridge and Griffiths 2002), and *gfp* encoding green fluorescent protein (Oda et al. 2004; Piuri et al. 2009). Alcaine et al. (2015) developed genetically engineered phage T7 (phage T7-ALP) carrying *phoA* that results in over-expression of alkaline phosphatase after infection of *E. coli* (Alcaine et al. 2015). Phage T7-ALP provides a promising opportunity but background noise could be an issue since alkaline phosphatase is a common enzyme found in a vast array of organisms including bacteria, fungi, plants, and animals (McComb et al. 1979; Alcaine et al. 2015). Moreover, the previous research on detection of bacteriophage-mediated alkaline phosphatase production was based on enzymatic essay using substrates that yield soluble products which distribute homogeneously in the solution resulting in a diluted signal (Alcaine et al. 2015); therefore, the signal-to-noise ratio may be reduced in the presence of food.

In order to overcome this obstacle, instead of colorimetric and chemiluminescent soluble substrates, our research focused on heterogeneous detection using an alkaline phosphatase substrate 2-(5′-chloro-2-phosphoryloxyphenyl)-6-chloro-4(3H)-quinazolinone (ELF-97) that, when hydrolyzed, gives a precipitated fluorescent product that localized at the site of reaction. Deposition of the precipitated ELF-97 product allows concentration of the signal which results in higher signal-to-noise ratio and increased detection sensitivity. ELF-97 is a non-fluorescent water-soluble substrate that once cleaved with alkaline phosphatase will yield the product, ELF-97 alcohol, that precipitates at the site of the reaction (Huang et al. 1993; Telford et al. 1999; Duhamel et al. 2009). This outstanding feature of ELF-97 results in signal deposition allowing detection of enzyme activity at the single cell level within few minutes after the reaction (González-Gil et al. 1998). ELF-97 has been applied for detecting mRNA in situ hybridization (Paragas et al. 1997), cytological labelling and imaging of mammalian cells (Paragas et al. 2002), and detection of alkaline phosphatase activity in marine planktons at the single-cell level (González-Gil et al. 1998; Nedoma et al. 2003; Peacock and Kudela 2012). To the best of our knowledge, none of the previous studies have investigated the application of ELF-97 for microbial detection approaches in food systems.

With the benefit of phage T7-ALP to induce alkaline phosphatase over-expression in *E. coli* and the potential of ELF-97 to endogenously detect a small amount of alkaline phosphatase enzyme, this research aims to develop a novel detection method of *E. coli* in authentic beverages samples. With fluorescence imaging and image analysis, we hypothesized that this approach will provide a method able to detect low concentrations of *E. coli* in a variety of different beverages matrices within less than 6 h.

## Materials and methods

### Bacteriophage and bacterial strains

Engineered bacteriophage T7-ALP was kindly provided by Dr. Sam Nugen (Cornell University). Bacteriophage T7-ALP has been amplified by inoculation of phage 10^5^ PFU/ml into 10^8^ CFU/ml of log-phase *E. coli* BL21 followed by 15 min of incubation at 37 °C for initial infection and 10 min of centrifugation at 16,100×*g* to harvest infected bacteria. Bacteria pellet was resuspended in 15 ml of sterile tryptic soy broth (TSB, Sigma-Aldridge, St. Louis, MO, USA) before incubation at 37 °C with 200 rpm constant shaking for further infection. After there was no visible turbidity, 3 ml of chloroform was added and the mixture was kept at 4 °C overnight to lyse intact cells. To separate cell debris, the mixture was centrifuged at 16,100×*g* for 10 min. The upper liquid phase which contains free phage was collected and centrifuged at 16,100×*g* for 10 min. The supernatant was discarded and the phage pellet was resuspended in sterile phosphate buffer saline (Fisher Scientific, Pittsburg, CA, USA) and stored at 4 °C. The titer of the phage stock was enumerated by standard plaque counting assay.

The model bacteria for this research was *E. coli* BL21 (ATCC BAA-1025) obtained from American type culture collection. Two strains of bacteria were used as controls: *Listeria innocua* (ATCC 33090) kindly provided by Dr. Linda Harris (University of California, Davis) and *Pseudomonas fluorescens* (ATCC 13525) from American type culture collection. All bacterial strains were stored in TSB containing 15% (vol/vol) glycerol at − 80 °C. For short-term storage, the glycerol stock of bacteria was streaked onto tryptic soy agar (Sigma-Aldridge, St. Louis, MO, USA) plates then incubated overnight at 37 °C for *E. coli* and at 30 °C for *Listeria innocua* and *Pseudomonas fluorescens*. The agar plates were then stored at 4 °C for further experiment.

### Beverage samples preparation

Coconut water (Vita coco, 100% coconut water) and apple juice (Signature select, 100% apple juice) were purchased from the local grocery store. Prior to the experiment, coconut water was filtered through a 0.22-micron syringe filter. To support the growth of bacteria and phage infection, TSB with double concentration was added to coconut water and apple juice at the ratio of 1:1. The pH of apple juice-TSB mixture was adjusted to 7 using 1 M tris before filtering through a 0.22-micron syringe filter.

### Bacterial inoculation, enrichment, and phage infection

For overnight culture preparation, a single colony of bacteria from agar plate was transferred to 10 ml of TSB and incubated at 37 °C with constant shaking at 200 rpm for 16 h. For inoculum preparation, 1 ml of the overnight culture (10^9^ CFU/ml) was centrifuged at 16,100×*g* for 1 min then the cell pellet was washed twice by resuspending in 1 ml of sterile phosphate buffer saline. The cell suspension was serial diluted and inoculated in 10 ml of TSB and beverages-TSB mixture; coconut water and apple juice, to the final concentration of 10^2^ and 10^3^ CFU/ml and incubated at 37 °C with constant shaking (200 rpm) for 4 h. Bacteriophage T7-ALP was added to the mixture to the final concentration of 10^6^ PFU/ml and incubated in the same condition for 15 and 30 min.

### Filtration and optical detection of alkaline phosphatase activity

To capture the infected bacterial cells, all 10 ml of the mixtures were filtered through 0.22-micron white polycarbonate membrane discs with a 19 mm diameter (Nucleopore Polycarbonate Whatman). After filtration, the filters were stained with ELF-97 alkaline phosphatase substrate using ELF™ 97 Endogenous Phosphatase Detection Kit (Molecular Probes, Eugene, OR, USA). The reaction mixture was prepared by diluting the ELF-97 phosphatase substrate 1:20 in ELF-97 developing buffer (provided in the kit) then spotted 20 µl of the reaction mixture on a microscopic slide. The filters were removed from a vacuum filtration system and put to the reaction mixture on the slides then another 20 µl of the reaction mixture was added on the top of the filter and spread evenly on the filter surface using sterile pipette tips. The slides with filters were incubated at room temperature in the dark for 30 min. To stop the enzymatic reaction, the filters were removed from the slides and put on the cellulose filter paper saturated with 1% formaldehyde for 5 min. All filters were counterstained with red fluorescent nucleic acid stain SYTO 60 (Molecular Probes, Eugene, OR) to stain all bacterial cells present on the filter. The ELF-97-stained filters were placed on the microscopic slides pre-spotted with 10 µl of 20 µM SYTO 60. Another 10 µl SYTO 60 was dropped on top of the filters and covered with cover glasses then observed under the Leica TCS SP8 STED 3× confocal microscope (Leica Microsystems, Mannheim, Germany) equipped with a white light laser for excitation. ELF-97 was excited by 405 nm STED laser and the fluorescent emission was collected at 452–560 nm. SYTO 60 was excited at 647 nm white light laser and the fluorescent emission was collected at 654–752 nm. All images were taken with a 100× oil-immersion objective (NA 1.4) with the laser power 5%. For negative control, the experiment was performed as described earlier with *E. coli* BL21 without adding phage and *L. innocua* and *P. fluorescens*. Experiments for all conditions were performed in triplicates.

### Image analysis for mean fluorescence intensity

Image analysis was performed using ImageJ (available for download at https://imagej.nih.gov/ij/ download.html). All images were binarized to have images of cells in black and background in white. The intensity threshold for each image was set at 70 when the maximum intensity was 225. The mean density of each cell particle was obtained using ImageJ’s particle analysis package. To eliminate the noise background signal, only particles with the size larger than 0.5 pixels were analyzed. The analysis of multiple images was repeated for each condition (N = 15).

### Alkaline phosphatase assay using FDP soluble fluorescent substrate

Fluorimetric detection of alkaline phosphatase was performed using Amplite™ Fluorimetric Alkaline Phosphatase Assay Kit (Green Fluorescence) (AAT Bioquest, Biomol, Hamburg, Germany). *E. coli* BL21 with the concentration of 10^2^, 10^3^, and 10^4^ CFU/ml were inoculated in 1 ml of beverage samples-TSB mixture before incubation at 37 °C with constant shaking (200 rpm) for 4 h. For phage infection, 10^6^ PFU/ml of T7-ALP phage was inoculated then incubated at the same condition for 30 min. The mixtures were centrifuged (16,100×*g*) for 10 min to separate the cell debris. Fifty microliters of the samples were mixed with the FDP substrate provided from the kit in a black flat-bottom 96-well plate. The fluorescence was measured using a microplate reader TECAN SpectraFluor Plus (TECAN Austria GmbH, Grödig, Austria) with excitation at 488 nm and emission at 520 nm. The experiments were performed in parallel with the blank which is 1 ml of beverage sample without *E. coli* and phage inoculation and negative controls of *E. coli* without phage infection.

### Statistical analysis

All experiments were done in triplicate independently for each condition. For image analysis, the total number of 15 images were analyzed (N = 15). The mean and standard deviation values were calculated within the samples in all cases. The Tukey’s HSD test was performed on the mean intensity of the fluorescent images and the signal/noise ratio of the fluorometric assay of alkaline phosphatase using FDP substrate in order to define the limit of detection. All statistical analysis was done using the R software.

## Results

### Schematic diagram of bacterial detection methods based on T7-ALP phage infection

**Fig. 1:**
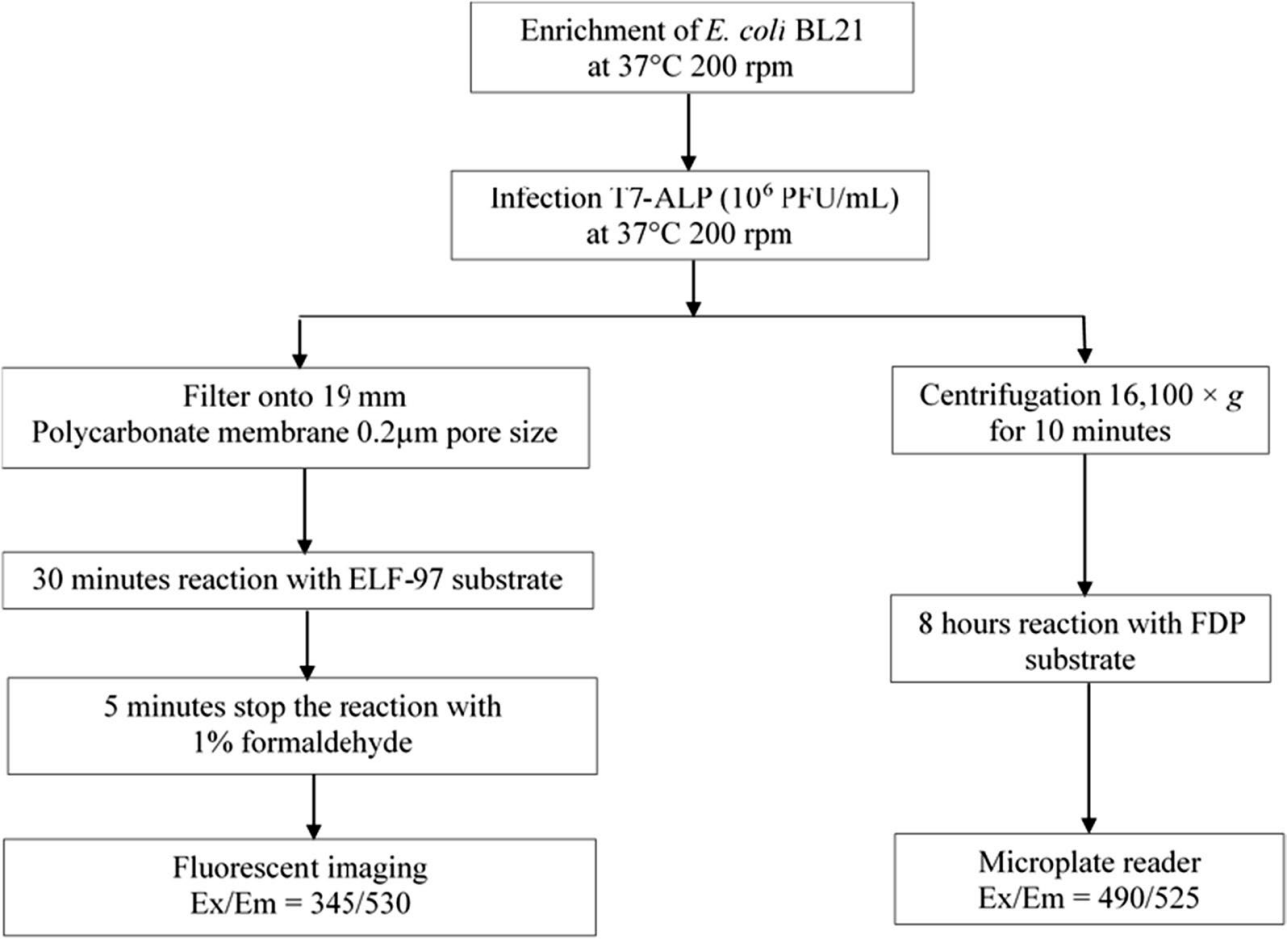
Schematic diagram of detection protocol based on alkaline phosphatase activity assay using soluble alkaline phosphatase substrate (FDP) and insoluble alkaline phosphatase substrate (ELF-97)

The protocols of *E. coli* detection based on the assay of phage-induced alkaline phosphatase activity using soluble and precipitated fluorescent substrates are shown in Fig. 1. Both methods started with 4 h of enrichment of *E. coli*. The initial concentration of *E. coli* in both beverage samples were 10^2^ CFU/ml and 10^3^ CFU/ml. After 4 h of enrichment, engineered phage T7-ALP was added to the sample allowing phage infection and expression of alkaline phosphatase. Both methods focus on detection of phage-induced alkaline phosphatase activity. The first method involves filtration to capture bacterial cells on the filter and detection of phage-induced alkaline phosphatase using ELF-97 substrate which is cell permeable and yields bright precipitated fluorescent product that can be visualized under the fluorescent microscope. Another approach is based on using FDP alkaline phosphatase substrate to detect alkaline phosphatase activity. The soluble fluorescent product of FDP was detected by measuring fluorescent signal using a microplate reader.

### Optical detection of *E. coli* using ELF-97 fluorescent substrate in TSB

Before investigating the detection method in authentic beverage samples, detection of *E. coli* in TSB was first performed. In 10 ml of sterile TSB, 10^2^ CFU/ml and 10^3^ CFU/ml were inoculated and incubated at 37 °C for 4 h with constant shaking at 200 rpm. T7-ALP phage with the final concentration of 10^6^ PFU/ml was added then the mixture was incubated at the same condition for 15 and 30 min. Ten milliliters of the mixture were filtrated onto a 0.2 µm white polycarbonate filter. *E. coli* BL21 in the same concentrations but without phage infection served as negative controls. Detection of alkaline phosphatase activity was performed by adding ELF-97 substrate directly onto bacterial cells captured on the filter. After counterstaining with syto60, the filters were observed under a confocal fluorescent microscope.

**Fig. 2:**
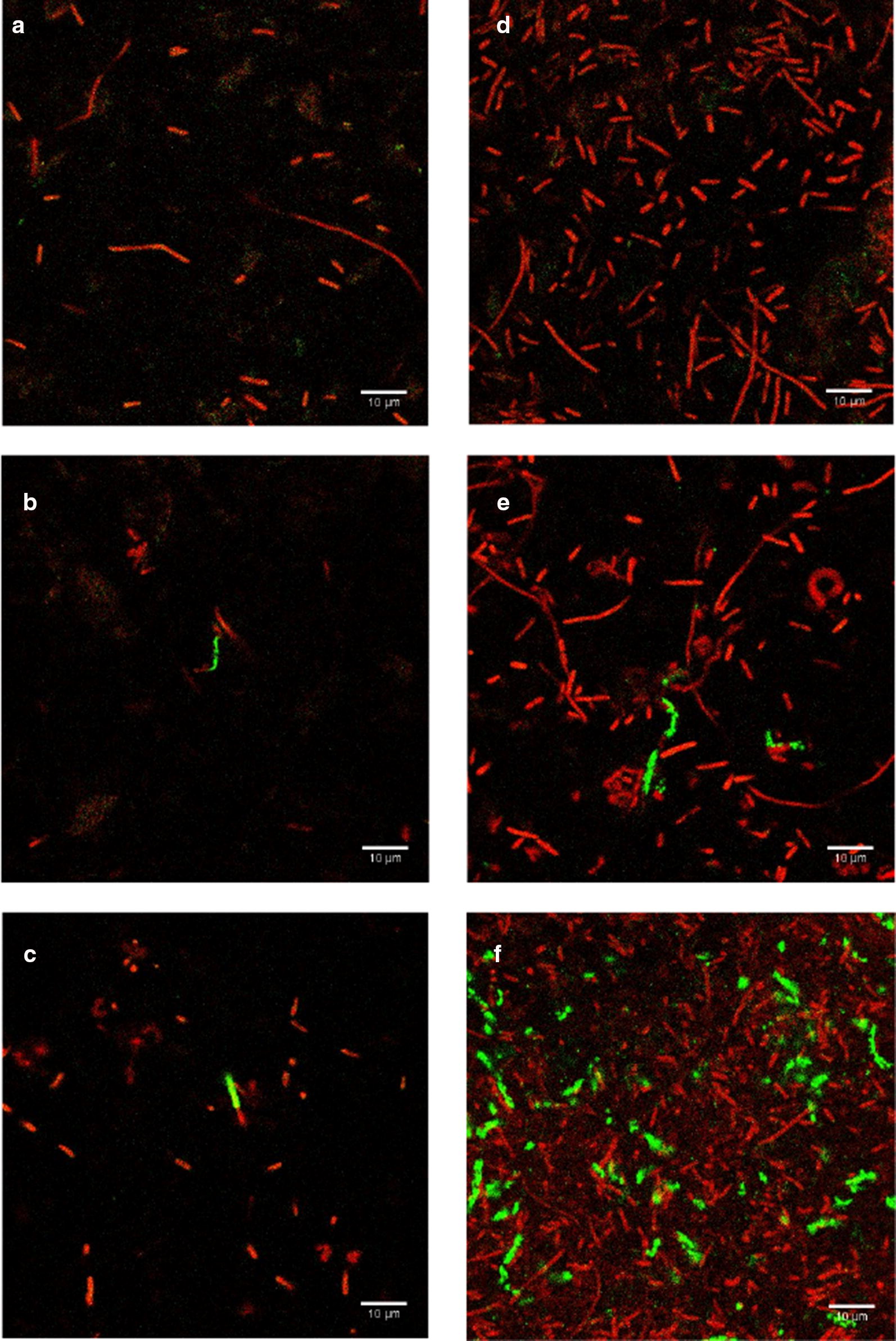
Representative fluorescent images of *E. coli* BL21 10^2^ CFU/ml after 4 h enrichment in TSB: **a** no phage infection, **b** after 15 min of T7-ALP infection, **c** after 30 min of T7-ALP infection. Fluorescent images of *E. coli* BL21 10^3^ CFU/ml after 4 h enrichment in TSB: **d** no phage infection, **e** after 15 min of T7-ALP infection, **f** after 30 min of T7-ALP infection

Representative fluorescent images are illustrated in Fig. 2. The total bacterial cells present on the filter appeared in red due to SYTO-60 red fluorescent nucleic acid stain. Without phage infection, Fig. 2a, d reveal that *E. coli* has no alkaline phosphatase activity after 4 h of enrichment without phage infection at a concentration of 10^2^ CFU/ml and 10^3^ CFU/ml, respectively. Figure 2b, c represent the fluorescent images of *E. coli* cells after 15 and 30 min of infection by phage T7-ALP when the initial bacterial concentration was 10^2^ CFU/ml. At both time points, at least one *E. coli* cell cluster per image was observed to have fluorescent signal localized inside the cell showing that the cell was infected and alkaline phosphatase was produced but the cell was not yet lysed by the phage. Figure 2e, f represent images of *E. coli* cells after 15 and 30 min of infection by phage T7-ALP when the initial bacterial concentration was 10^3^ CFU/ml. After 15 min of phage infection, few cells exhibit green fluorescence as shown in Fig. 2e but after the infection continue to 30 min, a representative image shows an increase in the number of cells that exhibit green fluorescence as show in Fig. 2f.

### Evaluation of issue of potential false positive identification of *E. coli*

**Fig. 3:**
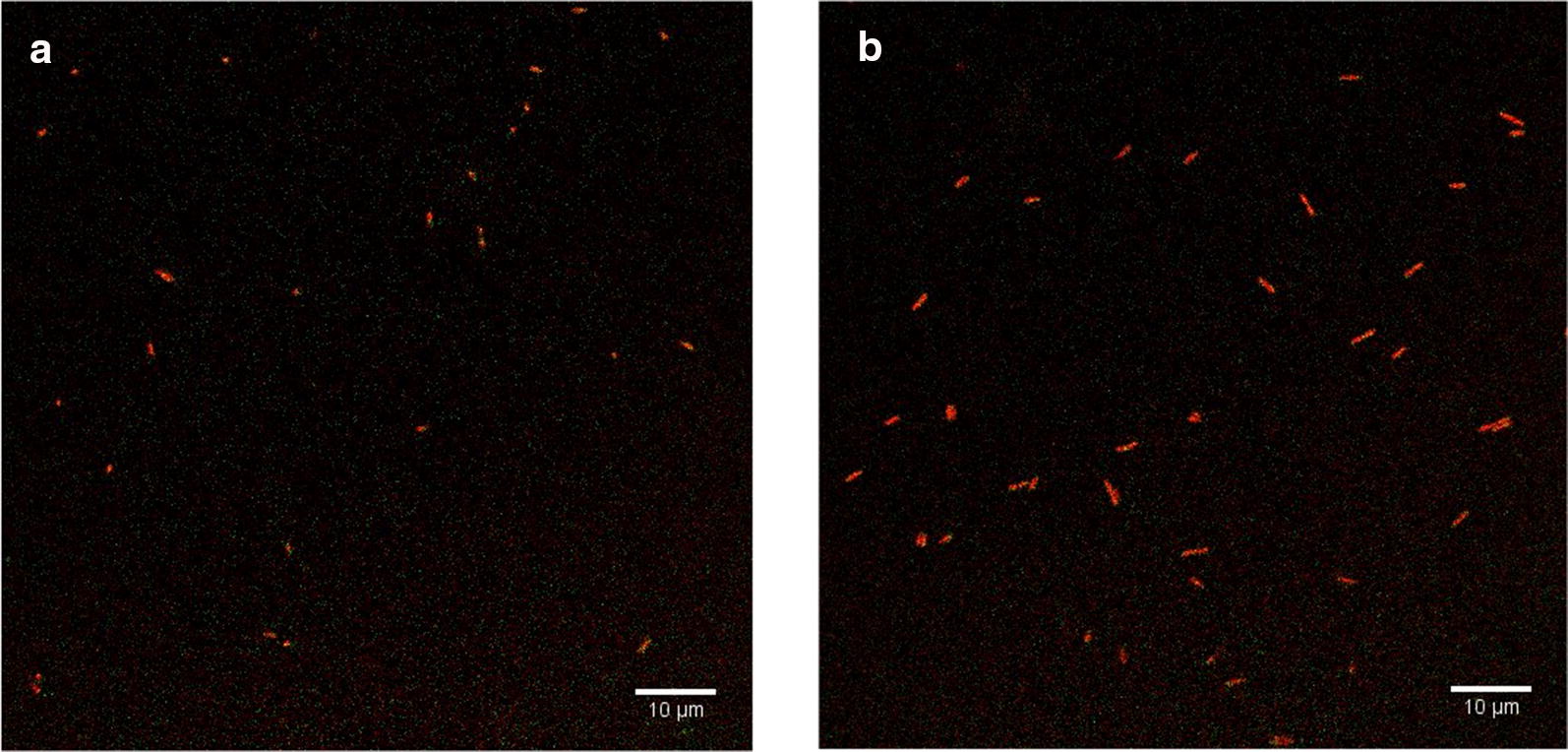
*L innocua* (**a**), *P. fluorescens* (**b**) after 4 h enrichment in TSB and 30-min infection with bacteriophage T7-ALP

Phage T7 infection is specific to *E. coli*; therefore, phage T7 will not be able to infect other bacterial strains or species thereby eliminating false positive results if deployed as a bacterial detection method. However, to more rigorously investigate this assumption, the specificity of this phage approach to *E. coli* detection was challenged using two food related bacteria: *L. innocua* and *P. fluorescens*. Both strains with an initial concentration of 10^3^ CFU/ml were enriched in TSB for 4 h before infection with 10^6^ PFU/ml of phage T7-ALP for 30 min. After filtration and reaction with ELF-97, there was no significant green fluorescent signal was detected (Fig. 3).

### Optical detection of *E. coli* in realistic challenging beverage matrices

**Fig. 4:**
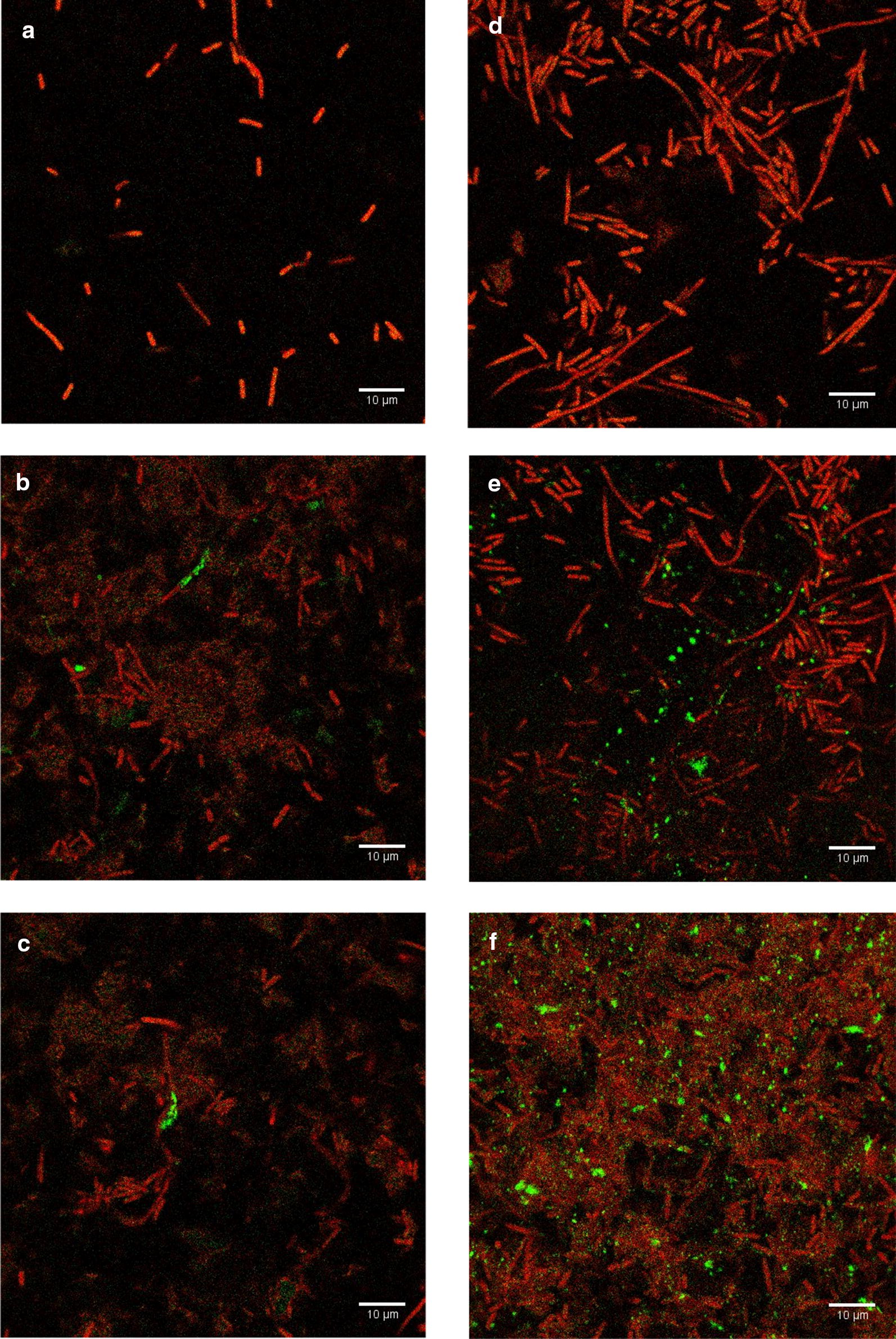
Representative fluorescent images of *E. coli* BL21 10^2^ CFU/ml after 4 h enrichment in coconut water-TSB mixture: **a** no phage infection, **b** after 15 min of T7-ALP infection, **c** after 30 min of T7-ALP infection. Fluorescent images of *E. coli* BL21 10^3^ CFU/ml after 4 h enrichment in coconut water-TSB mixture: **d** no phage infection, **e** after 15 min of T7-ALP infection, **f** after 30 min of T7-ALP infection

This detection method was also studied for detection of *E. coli* in an authentic complex beverage matrix. First, coconut water was examined, which predominantly contains sugar and minerals (Prades et al. 2012). The coconut water was filtered through 0.22 µm syringe filter to ensure no contamination of other bacteria then mixed with double-concentrated TSB to support bacterial growth of inoculated bacteria. *E. coli* with the concentration of 10^2^ CFU/ml and 10^3^ CFU/ml was inoculated into the coconut water-TSB mixture and incubated at 37 °C with 200 rpm constant shaking for 4 h for enrichment. For phage infection, 10^6^ PFU/ml was added and co-incubated at the same condition for 15 and 30 min. The mixtures were filtered through 0.2 µm white polycarbonate membrane before adding ELF-97 substrate and looked under the microscope. Representative fluorescent images were shown in Fig. 4. Figure 4a, d show the fluorescent images of 10^2^ CFU/ml and 10^3^ CFU/ml of *E. coli,* respectively. After 4 h of enrichment with no phage infection, the images show total cells with red fluorescence without visible green fluorescence of cell or background noise indicating that coconut water-TSB does not induce alkaline phosphatase production by *E. coli*.

Figure 4b illustrates fluorescent image of bacteria 10^2^ CFU/ml of *E. coli* after 4 h of enrichment and 15 min of phage T7-ALP infection. There was at least one cluster of cells per image that has green fluorescence of ELF-97 product. Even though the infection time was extended to 30 min, the number of cells with alkaline phosphatase activity did not increase. This result is similar to the result observed for phage T7-ALP infection of *E. coli* grown in TSB (Fig. 2).

In the case of *E. coli* 10^3^ CFU/ml, phage T7-ALP infection occurred a significant green fluorescent signal was detected. Interestingly, the signal occurred as within multiple small particles scattered outside the cells as can be seen in Fig. 4e. After the infection continued to 30 min, there was an increase in number of small punctate green fluorescence particles (Fig. 4f).

**Fig. 5:**
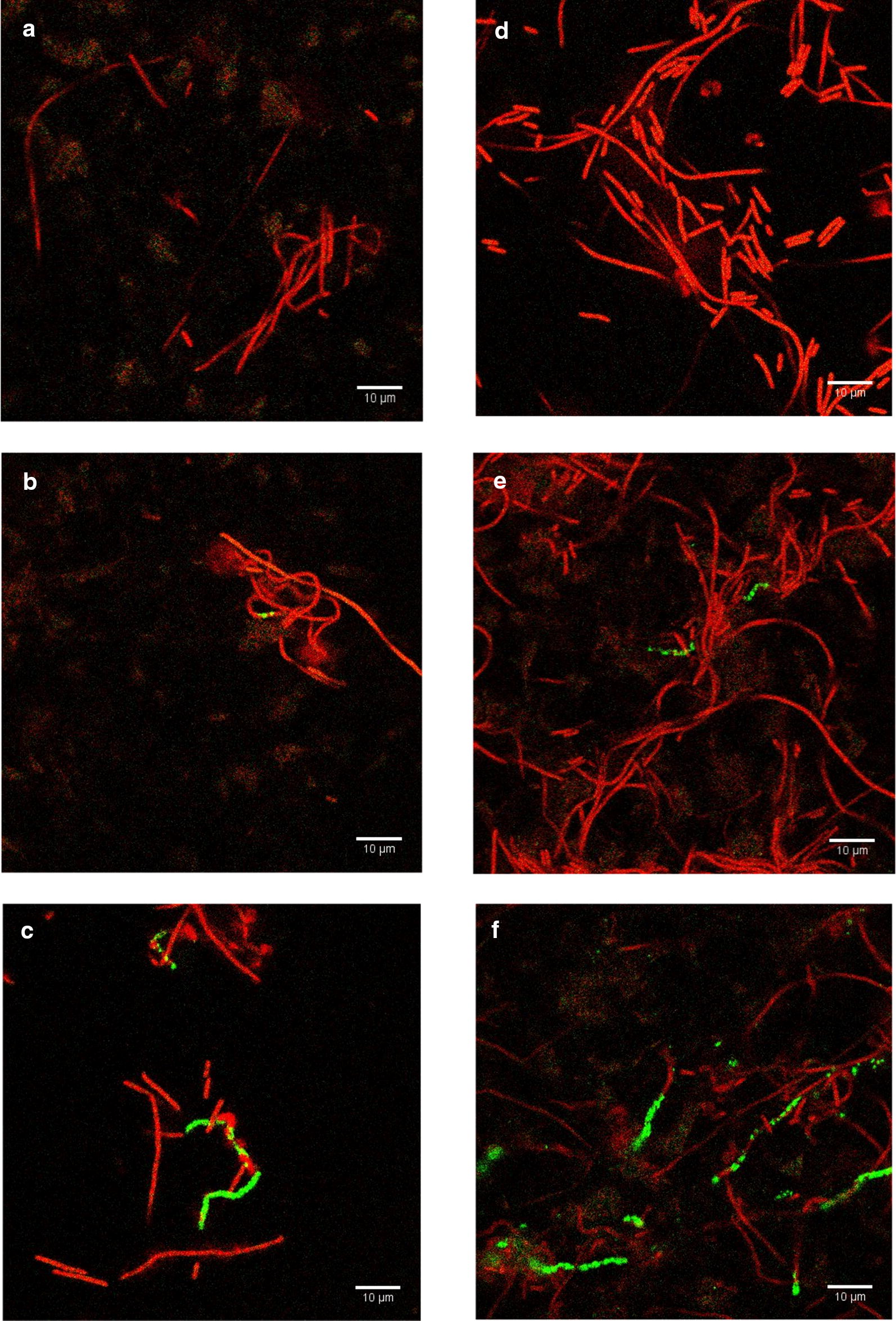
Representative fluorescent images of *E. coli* BL21 10^2^ CFU/ml after 4 h enrichment in apple juice-TSB mixture: **a** no phage infection, **b** after 15 min of T7-ALP infection, **c** after 30 min of T7-ALP infection. Fluorescent images of *E. coli* BL21 10^3^ CFU/ml after 4 h enrichment in apple juice-TSB mixture: **d** no phage infection, **e** after 15 min of T7-ALP infection, **f** after 30 min of T7-ALP infection

As an alternative beverage sample, apple juice was tested for *E. coli* using this methodology. Apple juice predominantly has sugars and minerals but has an acidic pH less than 6. *E. coli* contaminated apple juice was used to set up an enrichment. An apple juice-TSB mixture was adjusted to pH 7 before filtration through 0.22-micron syringe filter and bacterial inoculation. The detection method using engineered phage T7-ALP and alkaline phosphatase substrate ELF-97 was performed as described earlier. Representative fluorescent images of *E. coli* in apple juice-TSB after reaction with ELF-97 are shown in Fig. 5. Figure 5a, d show the results of *E. coli* after 4 h of enrichment without phage infection with initial concentration of *E. coli* of 10^2^ CFU/ml and 10^3^ CFU/ml, respectively. After 4 h of enrichment, the bacteria appeared to have filamentous morphology; however, there was no cell with visible green fluorescent signal from precipitated ELF-97 fluorescent product.

Following 15 min of phage T7-ALP infection, images of bacteria originating from a concentration of 10^2^ and 10^3^ CFU/ml were observed as shown in Fig. 5b, e, respectively. This revealed green fluorescent signal marking the location of significant alkaline phosphatase activity and the signals were very bright and easy to distinguished from the background. When the infection was extended to 30 min prior to imaging, there was more than one cell with green fluorescence and the signal became more intense and appeared along the filamentous arrangement, Fig. 5c, f. For Apple juice, signal was always confined to cells, with no apparent small green fluorescent particle, suggesting that in this experiment situation there was no loss of bacterial cell integrity.

### Image analysis of T7-ALP infected *E. coli* fluorescent images

**Fig. 6:**
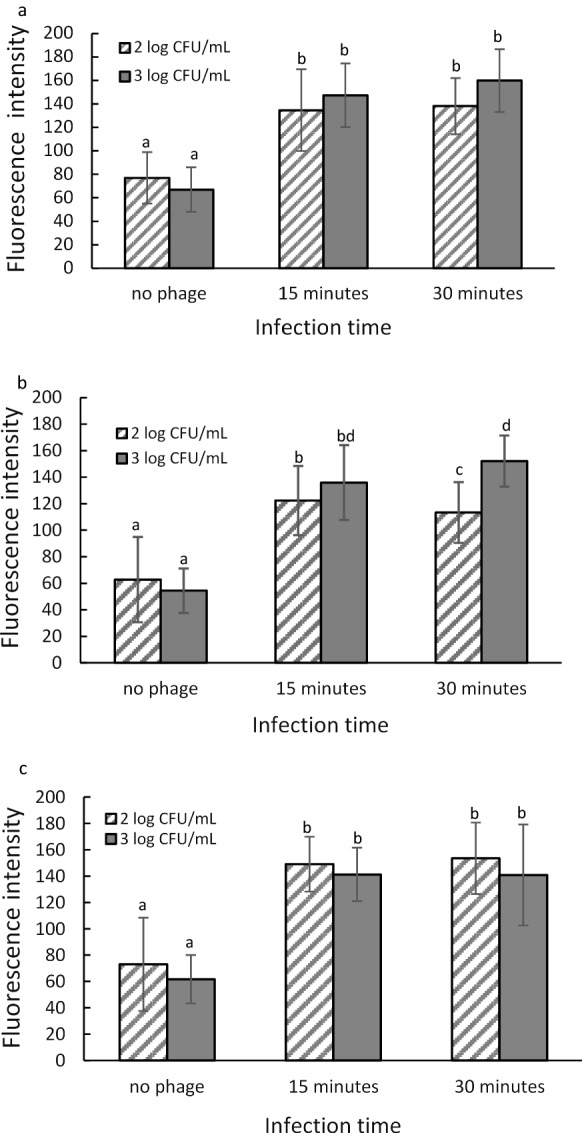
Mean gray value of fluorescent images of *E. coli* BL21 10^2^ CFU/ml and 10^3^ CFU/ml 4 h enrichment in TSB (**a**), coconut water (**b**), and apple juice (**c**) after 15- and 30-min infection with bacteriophage T7-ALP. Treatments with different letters are significantly different (P < 0.05). Error bars indicate ± standard deviation of means

Figure 6a–c show the level of mean intensity of the fluorescent signal of ELF-97 precipitated fluorescent product in TSB, coconut water-TSB mixture, and apple juice-TSB mixture, respectively. In all media, initial bacteria concentration of 10^2^ CFU/ml and 10^3^ CFU/ml gave the significantly higher mean intensity compare to negative control which was bacteria with no phage infection. However, there is no significant difference (*P* < 0.05) between the mean intensity of 10^2^ CFU/ml and 10^3^ CFU/ml. After 30 min of infection in coconut water, the result indicates a decrease in the mean fluorescence intensity. This is probably due to the fact that, in coconut water, after 30 min of infection most of bacterial cells were lysed causing the release of alkaline phosphatase thus the signal was no longer localized in bacterial cells. Therefore, it is critical that the bacteria-phage mixture was filtered before lysis of bacterial cells in order to maintain the high intensity of the fluorescent signal.

### Detection of *E. coli* based on alkaline phosphatase activity using a soluble FDP fluorescent substrate

**Fig. 7:**
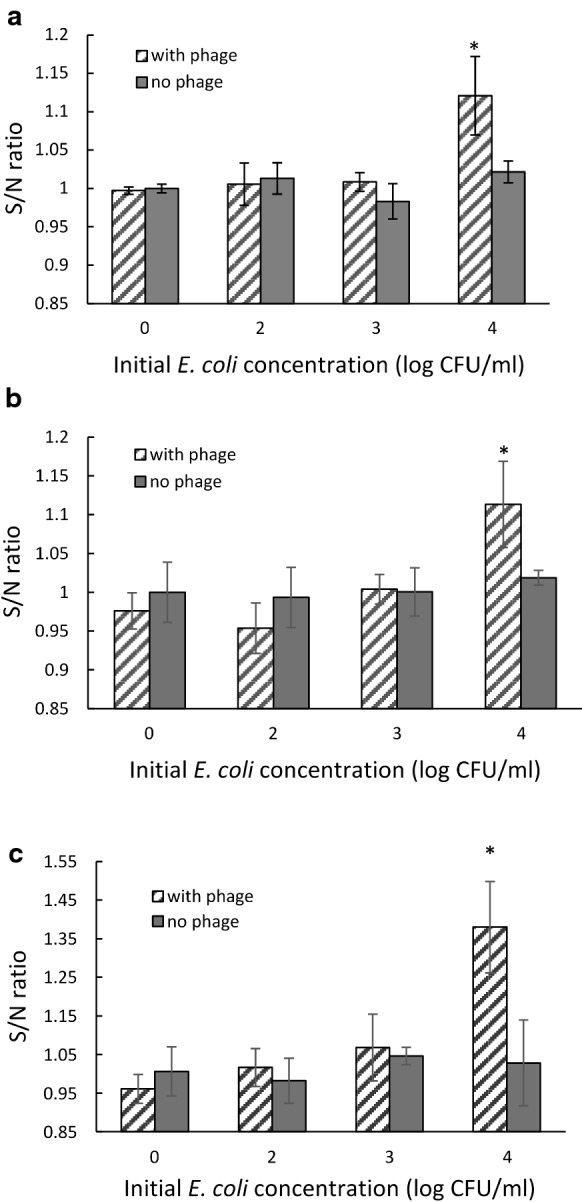
Signal to noise ratio of Alkaline phosphatase over-expressed by *E. coli* BL21 after 4 h of enrichment in TSB (**a**), coconut water (**b**), and apple juice (**c**) after infection with phage T7-ALP for 30 min. Asterisk indicates significant difference (P < 0.05) from negative control. Error bars indicate ± standard deviation of means

Signal-to-noise ratio of the fluorescent signal when infection took place in TSB, coconut water-TSB mixture, and apple juice-TSB mixture are shown in Fig. 7a–c, respectively. In TSB, the detection limit of *E. coli* was 10^4^ CFU/ml after 4 h of reaction with FDP substrate. For detection in beverage sample, initial cell concentration of 10^4^ CFU/ml in coconut water-TSB mixture can be detected after 8 h of reaction, and after 12 h in apple juice-TSB mixture while there was no significant difference between negative control of no bacteria and *E. coli* 10^3^ and 10^2^ CFU/ml. To detect 10^4^ CFU/ml, it requires the reaction time of 4 h, 8 h, and 12 h in TSB, coconut water, and apple juice, respectively.

## Discussion

Bacteriophage has been continuously employed as the valuable tool for bacteria detection due to its specificity and rapidity of the infection cycle, robustness, and low-cost preparation (Richter et al. 2018). Bacteriophage T7 and its host, *E. coli*, is one on the most widely studied models for bacteria detection. The simplicity of bacteriophage T7 genome has enabled genetically engineering for insertion of the reporter gene. Several bacteriophage-based bacteria detection approaches have been recently implemented in order to improve bacteria detection sensitivity (Table 1). These previous studies developed the detection methods based on colorimetric, bioluminescent, and electrochemical detection of enzymatic activity using substrates that yield soluble products; therefore, their detection sensitivity relies on the cell concentration and/or immobilization of the reporter enzyme on biomaterial substrates.

**Table 1:** Short summary of bacteriophage-based rapid bacteria detection studies using genetically engineered bacteriophage T7

Method description	Detection limit	Assay time	Matrices	Potential limitations	References
Detection of T7-induced alkaline phosphatase activity using chemilumiscent methods	10^3^ CFU/ml	6 h	Luria Broth	Detection of enzymatic activity can be affected by background noise	Alcaine et al. (2015)
Detection of phage T7 amplification using lateral flow assays with phage-based enzymatic reporter	10^2^CFU/100 ml	9 h	River water	Reduced detection efficacy in complex matrices may occur due to non-specific binding to antibody	Alcaine et al. (2016)
Detection of T7-induced β-galactosidase using colorimetric substrate	10^2^ CFU/ml	7 h	Drinking water, skim milk, orange juice	Additional cost and complexity due to lyophilization	Chen et al. (2017)
Detection of T7-induced β-galactosidase using electrochemical method	10^2^ CFU/ml	7 h	Drinking water, skim milk, orange juice	Low sample volume (1 ml)	Wang et al. (2017)
Detection of T7-induced luciferase and alkaline phosphatase by filter-based colorimetric and bioluminescence method	1 CFU/100 ml	10 h	Drinking water	Reduced detection efficacy in complex matrices may occur due to background color and large food particles	Hinkley et al. (2018b)
Detection of T7-induced luciferase immobilized on microcrystalline cellulose	< 10 CFU per 100 ml	3 h	Drinking water	Reduced detection efficacy in complex matrices may occur due to non-specific binding to cellulose	Hinkley et al. (2018a)

This study demonstrates the novel approach of enhancing the detection sensitivity by detection of reporter enzyme using precipitated substrate followed by fluorescent imaging and image analysis. From the results we established that infection of engineered bacteriophage T7-ALP coupled with the use of ELF-97 as alkaline phosphatase substrate was a viable option that could be developed into a food safety application. Localization of the fluorescent precipitated signal inside the cell resulting in increased signal intensity allowing visualization of a single bacterial cell. Further, they are consistent with other studies. ELF-97 was used for detection of alkaline phosphatase activity as a phosphate stress marker in marine phytoplankton with fluorescent imaging and flow cytometry (González-Gil et al. 1998; Dyhrman 1999; Nedoma et al. 2003; Van Wambeke et al. 2008). ELF-97 yielded a highly sensitive detection of a single cell of phosphate-stressed marine bacteria (Duhamel et al. 2009). Huang et al. (1998) successfully used ELF-97 to detect alkaline phosphatase expression of bacteria colonies and biofilm in phosphate starvation condition (Huang et al. 1998).

Developing a robust pathogen detection system demands that the method is ultimately specific without unexpected false positive results. As bacteriophage is highly specific to bacterial host strain, other bacterial strain will not be infected by bacteriophage T7-ALP and will not overexpress alkaline phosphatase thus no fluorescent signal detected. Two strains of foodborne bacteria, *L. innocua and P. fluorescens,* were tested to evaluated the detection specificity. *L. innocua* is a nonpathogenic surrogate of *Listeria monocytogenes* which is a Gram-positive foodborne pathogenic bacterium commonly found in food and agricultural product (Friedly et al. 2008). While *P. fluorescens* is representing Gram-negative bacteria with rod shape that are commonly found in food system to cause problems with spoilage (Rajmohan et al. 2002). The results indicated no significant green fluorescent signal was detected which reinforced the potential to distinguish target bacteria from other foodborne bacterial strain. Although it might not be strictly defined as a false positive, it remains possible that the resulting alkaline phosphatase activity observed by *E. coli* following phage T7-ALP infection is due to a stress condition resulting in induction of the endogenous *E. coli phoA*. To test this possibility 10^3^ CFU/ml of *E. coli* was infected by wildtype T7 phage. This alternative possibility of *phoA* expression was not supported by the experimental results, which showed no significant green fluorescent signal.

Detection of *E. coli* in the complex beverage matrices is the essential step to evaluate the effect of food component on bacteriophage infection and expression of *phoA* gene. When the 30-min infection took place in coconut water (Fig. 4f), green fluorescence particles were not in the cell shape yet appeared in small punctate particles. We interpret that these results show that some of the integrity of some infected cells were compromised which caused the release of alkaline phosphatase. Assuming loss of cell integrity is due to phage-mediated cell lysis following replication, then indicating that phage T7-ALP conditions provided by cultivation in coconut water-TSB may shorten the phage replicative cycle.

*Escherichia coli* cells, after 4 h of enrichment in apple juice, displayed filamentous morphology (Fig. 5) which may be due to the SOS response noted previously by other investigators (Justice et al. 2006). Apple juice contains high content of phenolic compounds which show antibacterial activity against *E. coli* (Kahle et al. 2005; Alberto et al. 2006), which may account for the induction of the filamentous cell structure. However, there was no cell with visible green fluorescent signal from precipitated ELF-97 fluorescent product showing that these apparently stressed conditions have impact on the expression of endogenous *E. coli phoA* and, therefore, no significant green fluorescent signal detected. After bacteriophage T7-ALP infection (Fig. 5b, c, e, f), the signal was confined to distinctive segments of the filamentous cellular arrangement, revealing that phage T7-ALP replicated at this timepoint within a subset of *E. coli* cells forming the filament.

Image analysis results indicated that green fluorescence can represent the activity of phage-induced alkaline phosphatase since the signal intensity of phage-infected bacteria is significantly different from the auto-fluorescent of uninfected bacteria. Infection time has no influence on alkaline phosphatase activity when the media were TSB and apple juice as there is no changes in the mean fluorescence intensity. However, there was a decrease in fluorescence intensity after 30 min of infection in coconut water which may due to the release of alkaline phosphatase after cell lysis. This finding indicated that the natural properties and compositions of food matrices may influence the infection rate of bacteriophage which results in different optimal infection time for different food samples.

For detection of alkaline phosphatase activity using soluble substrate, FDA, the length of reaction time depends on the concentration of enzyme in the solution. Since the infection time is 30 min, the cells were not completely lysed; therefore, phage-mediated alkaline phosphatase was not released to the solution. Unlike the signal of precipitated fluorescent substrate, the signal from soluble fluorescent substrate is dissolved in the solution resulting in lower detection sensitivity. The results also indicate the difference in reaction time required to detect 10^4^ CFU/ml in difference infection media. More complexion of media resulted in more time required for *E. coli* detection using this approach as the composition of coconut water and apple juice may interfere with the reaction of alkaline phosphatase.

Overall, the results of this study indicated that the detection of alkaline phosphatase activity induced by engineered bacteriophage infection using substrate that yields precipitated fluorescence product coupled with fluorescence imaging and quantitative image analysis is a promising approach for rapid and highly specific bacterial detection. This proof-of-concept system can be applied in the real complex beverage matrices.
